# Xylose-fermenting *Pichia stipitis* by genome shuffling for improved ethanol production

**DOI:** 10.1111/1751-7915.12092

**Published:** 2014-01-07

**Authors:** Jun Shi, Min Zhang, Libin Zhang, Pin Wang, Li Jiang, Huiping Deng

**Affiliations:** 1 Key Laboratory of Yangtze River Water Environment, Ministry of Education, College of Environmental Science and Engineering, Tongji UniversityShanghai, China; 2 Division of Cardiology, Shanghai Changning Central HospitalShanghai, China; 3 First People's Hospital of Yunnan ProvinceKunming, China

## Abstract

Xylose fermentation is necessary for the bioconversion of lignocellulose to ethanol as fuel, but wild-type *Saccharomyces cerevisiae* strains cannot fully metabolize xylose. Several efforts have been made to obtain microbial strains with enhanced xylose fermentation. However, xylose fermentation remains a serious challenge because of the complexity of lignocellulosic biomass hydrolysates. Genome shuffling has been widely used for the rapid improvement of industrially important microbial strains. After two rounds of genome shuffling, a genetically stable, high-ethanol-producing strain was obtained. Designated as TJ2-3, this strain could ferment xylose and produce 1.5 times more ethanol than wild-type *Pichia stipitis* after fermentation for 96 h. The acridine orange and propidium iodide uptake assays showed that the maintenance of yeast cell membrane integrity is important for ethanol fermentation. This study highlights the importance of genome shuffling in *P. stipitis* as an effective method for enhancing the productivity of industrial strains.

## Introduction

The development of bioethanol production has received growing interest. Xylose is the second most abundant monosaccharide after glucose in lignocellulose hydrolysates (Jeffries and Jin, 2004). High ethanol yields from lignocellulosic residues are dependent on the efficient use of all available sugars, including glucose and xylose. The efficient fermentation of xylose is required to develop economically viable processes for the production of bioethanol from lignocellulosic biomass. *Saccharomyces cerevisiae* is an important industrial working species for ethanol production because it can produce high-titre ethanol from hexose sugars and demonstrate high ethanol tolerance. However, *S. cerevisiae* cannot ferment xylose (Jeppsson *et al*., 2003). By contrast, *Pichia stipitis* is one of the best wild-type xylose-fermenting species, which can produce high ethanol yields from xylose (Jeffries *et al*., 2007; Agbogbo and Coward-Kelly, 2008). This species has a low tolerance to ethanol and sugar, which has restricted its use as an industrial strain for large-scale bioethanol production from xylose. Therefore, most research groups have focused on the metabolic engineering of *S. cerevisiae* and *P. stipitis* to enable xylose metabolism in either species for growth and ethanol production. However, metabolic engineering is tedious, labour-intensive and time-consuming.

The genome shuffling technique has the advantage of providing abundant random mutations at different positions on the entire genome without requiring genome sequencing data or network information. Thus, genome shuffling has advanced the construction of mutant phenotypes, as compared with the conventional protocols (Ness *et al*., 1999; Gong *et al*., 2009). This technique can be integrated with metabolic engineering to facilitate the creation of complex phenotypes, thereby increasing the metabolite yield (Patnaik *et al*., 2002; Dai and Copley, 2004). Genome shuffling has been successfully used to rapidly improve prokaryotic strains and eukaryotic cells (Zhang *et al*., 2012a). However, this strategy largely depends on the efficiency of protoplast fusion techniques. Several important factors have been tested, and the necessary conditions have been optimized for the protoplast preparation and regeneration of *P. stipitis* strains prior to protoplast fusion.

Huang and colleagues (2009) improved ethanol production in a *P. stipitis* strain via the fermentation of acid-hydrolysed rice straw. Bajwa and colleagues (2009) obtained an ultraviolet light (UV)-mutagenized strain that could ferment hardwood sulfite liquor. However, research on the cellulosic ethanol production of this strain remains limited. In the present study, we attempted to improve the ethanol productivity of xylose-fermenting *P. stipitis* by genome shuffling. The resulting mutant demonstrated increased ethanol tolerance. Finally, the mechanism for ethanol production improvement was investigated in this study.

## Results and discussion

### Preparation and regeneration of protoplasts

Genome shuffling was successfully used to rapidly screen various strains of prokaryotic and eukaryotic cells (Patnaik *et al*., 2002; Dai and Copley, 2004). This process largely depends on the efficiency of protoplast preparation and fusion. This study aimed to develop a rapid and reliable modified method of genome shuffling to generate a mutant strain with enhanced xylose fermentation. Consequently, we optimized some conditions for the protoplast preparation and regeneration of *P. stipitis* prior to protoplast fusion. The 16 h cultures of the yeast were incubated in 1% (w/v) β-mercaptoethanol and 2% (w/v) zymolyase for 60 min to digest the cell wall. The protoplast was suspended in a 10 ml test tube with the protoplast formation buffer (PB) as an osmotic stabilizer. The rates of protoplast preparation and regeneration were 90 ± 1% and 19 ± 2% respectively (Table 1). The high efficiency of protoplast preparation and regeneration effectively accelerated the strain mutation.

**Table 1:** The rates of protoplast preparation and regeneration

	Time (min)						
	30	40	50	60	70	80	90
Protoplast formation ratio (%)	77 ± 3%	85 ± 1%	90 ± 1%	83 ± 4%	80 ± 5%	75 ± 2%	65 ± 6%
Protoplast regeneration ratio (%)	13 ± 4%	17 ± 3%	19 ± 2%	16 ± 3%	15 ± 4%	12 ± 5%	10 ± 5%

Data are expressed as the mean values ± standard deviation of at least three independent experiments.

### Protoplast inactivation

Genome shuffling is a facile technique that has been used to improve the phenotypes of several industrial microorganisms using inactivated parental protoplasts. This method allows for the simultaneous recombination of several genomes at different sites without requiring detailed genomic information. Therefore, multiple recombination events and several gene mutants can rapidly and efficiently occur, thereby generating a large number of strains that can be screened for the desired phenotypes. During classical genome shuffling, wild-type strains are subjected to traditional mutagenesis processes (Zhao *et al*., 2008). However, when the initial protoplasts are inactivated via a single method, protoplast regeneration becomes difficult. Therefore, we chose different parent protoplast inactivation methods according to the principle of complementary protoplast damage and applied them in this study prior to protoplast fusion.

The protoplasts were inactivated by heating, UV exposure and UV exposure combined with LiCl treatment. For heating inactivation, the protoplasts were incubated in a 60°C water bath for 30 min, thereby resulting in a lethality of 100%. For UV inactivation, an irradiation time of 40 min resulted in 100% lethality. For LiCl treatment combined with UV inactivation, incubation in 0.6% (w/v) LiCl with exposure to UV irradiation for 30 min was considered the best condition for protoplast inactivation (Table 2).

**Table 2:** Protoplast susceptibility to different inactivation treatments over time

Heat (min)	5	10	15	20	25	30
Lethal rate (%)	18 ± 2	42 ± 3	64 ± 2	85 ± 6	98 ± 5	100 ± 0
UV (min)	10	15	20	25	30	40
Lethal rate (%)	50 ± 3	60 ± 4	70 ± 3	80 ± 6	90 ± 4	100 ± 0
UV + LiCl (min)	5	10	15	20	25	30
Lethal rate (%)	35 ± 4	50 ± 6	65 ± 3	73 ± 4	82 ± 5	100 ± 0

Data are expressed as the mean values ± standard deviation of at least three independent experiments.

### Protoplast fusion

**Figure 1:**
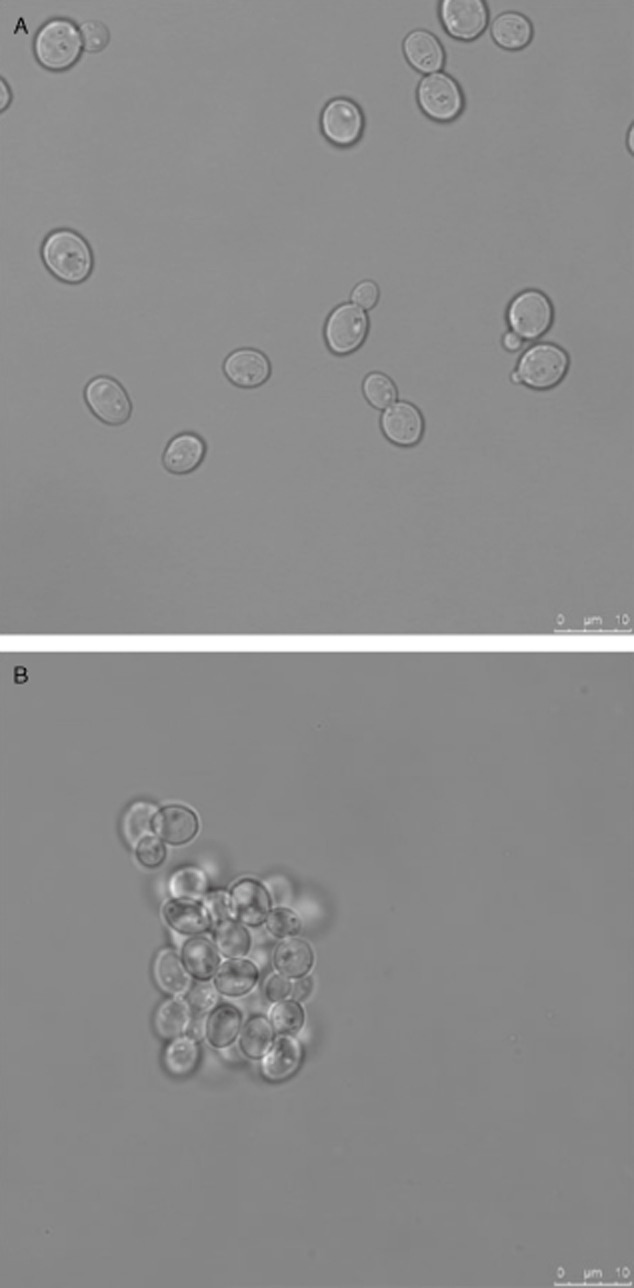
Protoplast fusion of genome-shuffled strains observed by CLSM.

High-efficiency protoplast fusion is essential for successful genome shuffling. Given the toxicity of polyethylene glycol (PEG), the fusion regeneration rate and consequently the fusion rate were critically decreased when the fusion time was too long or the PEG concentration was too high. Thus, the concentrations of PEG and CaCl_2_, as well as the fusion time, significantly influenced protoplast fusion efficiency in a previous study. Their data showed that the PEG concentration was the most important factor during protoplast fusion, followed by the CaCl_2_ concentration and the fusion time. Our optimized fusion conditions required 35% (w/v) PEG 6000, 10 mM CaCl_2_ and 30 min of fusion time (Table 3). Highly efficient fusion could generate more background mutations, which improved the genomic stability. Upon induction by PEG 6000, the protoplasts aggregated and fused together because of the increased cell membrane fluidity. The process of protoplast fusion was visualized by CLSM (Fig. 1).

**Table 3:** The results of experimental conditions used for fusion

	Factor			
Level	PEG concentration (%)	CaCl_2_ concentration (mM)	T (min)	Fusion rate (%)
1	25	5	20	0.02236
2	25	10	30	0.07315
3	25	15	40	0.10023
4	30	5	20	0.12686
5	30	10	30	0.05762
6	30	15	40	0.03476
7	35	5	20	0.04153
8	35	10	30	0.15836
9	35	15	40	0.08377

### Genome shuffling for screening of high ethanol production strains

Although traditional breeding methods have successfully selected for various microorganisms, such methods are time-consuming and expensive. Another limitation that must be overcome is the insufficient ethanol yield for sustaining bioethanol production, even though the wild-type strain could ferment xylose. This study attempted to use a rapid and reliable genome-shuffling method to develop a *P. stipitis* strain with improved xylose fermentation.

Patnaik and colleagues (2002) enhanced the acid tolerance of a *Lactobacillus* strain by genome shuffling, whereas Zhang and colleagues (2002) enhanced the antibiotic yield from a *Streptomycetes* strain. Yu and colleagues (2008) likewise used genome shuffling to successfully improve l-lactic acid production. The production of other biochemical products, such as taxol (Zhao *et al*., 2008), ethanol (Shi *et al*., 2002; Hou, 2010) and bioinsecticides (Jin *et al*., 2009), was definitely increased by genome shuffling. In this report, we demonstrated that genome shuffling could improve xylose metabolism in *P. stipitis*.

The protoplasts of *P. stipitis* were prepared and fused in the first step. The recombinant strains were selected on YNB with 5% xylose (YNBX) plates at 35°C for 2 days. Eight yeast strains (TJ1-1 to TJ1-8) rapidly grew on these plates, and their ethanol production after incubation at 30°C for 72 h was measured in YNBX. The mutant strain with the best ethanol production was TJ1-8 (Table 4). This potential strain was used as the parent strain for the second-round genome shuffling. After the second round of protoplast fusion, the mutant strain was cultured on the plates containing 6.8 g l^−1^ YNB, 5% xylose, 5% ethanol and 2% agar. Four positive colonies (TJ2-1 to TJ2-4) were obtained and their ethanol production was tested. The mutant (TJ1-1 to TJ1-8) and wild-type strains did not grow on the selective media, neither was additional ethanol produced. Thus, the potential recombinant strains TJ1 and TJ2, as well as the wild-type strain, of *P. stipitis* were evaluated for their xylose fermentation capability in 250 ml shake flasks filled with 100 ml of the fermentation medium with 5% xylose. All the results are listed in Table 4, with the TJ2-3 strain demonstrating enhanced ethanol production, as compared with the wild-type *P. stipitis* and the TJ1 mutants.

**Table 4:** The ethanol production from xylose of wild-type and mutant strains

Strain^a^	Xylose [%(w/v)]	Ethanol [%(w/v)]
Blank	4.48 ± 0.12	–
*P. stipitis*	0.77 ± 0.26	1.63 ± 0.14
TJ1-1	0.56 ± 0.33	1.74 ± 0.13
TJ1-2	0.53 ± 0.20	1.76 ± 0.11
TJ1-3	0.59 ± 0.33	1.71 ± 0.13
TJ1-4	0.55 ± 0.31	1.78 ± 0.11
TJ1-5	0.56 ± 0.31	1.79 ± 0.11
TJ1-6	0.49 ± 0.30	1.74 ± 0.10
TJ1-7	0.50 ± 0.34	1.75 ± 0.14
TJ1-8	0.39 ± 0.25	1.90 ± 0.15
TJ2-1	0.26 ± 0.32	2.17 ± 0.12
TJ2-2	0.29 ± 0.36	2.09 ± 0.13
TJ2-3	0.17 ± 0.12	2.26 ± 0.16
TJ2-4	0.22 ± 0.33	2.19 ± 0.13

a TJ1 and TJ2, and their parents, *P. stipitis* were inoculated in 100 ml of fermentation medium containing experiments.

### Fermentation of glucose, xylose and mixed sugars

**Figure 2:**
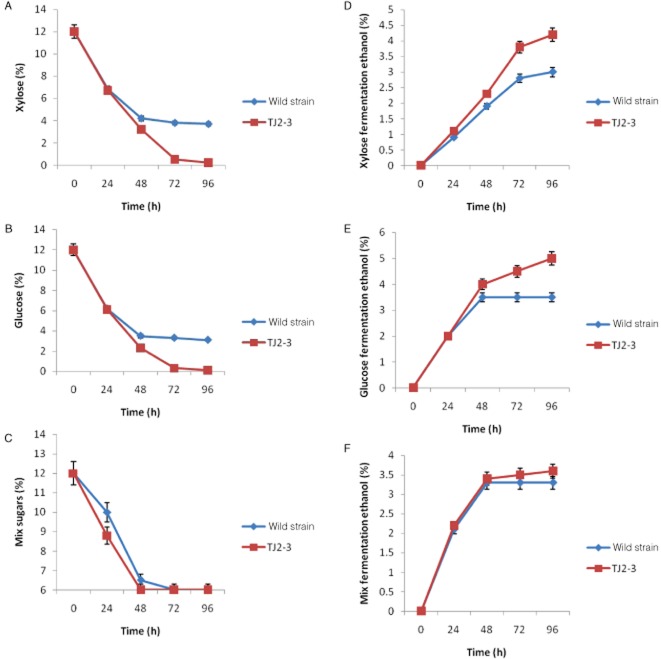
Time-course plot of ethanol production from the fermentation medium containing 12% xylose (A, D), 12% glucose (B, E) and 12% mixed sugars (C, F) by the *P. stipitis* wild-type (blue) and TJ2-3 (red) strains. Results are presented as the means of three independent experiments.

The fermentation capability of the wild-type and TJ2-3 strains was independently tested in the presence of different sugar conditions. The total sugar concentration was maintained at 120 g l^−1^ for all experiments, and the tests were conducted in duplicate. As shown in Fig. 2, the wild-type and TJ2-3 strains consumed both glucose and xylose. The TJ2-3 strain produced ethanol from xylose (Fig. 2A), glucose (Fig. 2B) and mixed sugars (Fig. 2C) at faster rates, with higher yields than the wild-type strain (Table 5). The TJ2-3 strain produced 4.4% and 4.8% ethanol from xylose and glucose alone, respectively, whereas the wild-type strain only produced approximately 3.2% and 3.6% ethanol. The ethanol production of the TJ2-3 strain was approximately 1.5 times higher than that of the wild-type strain.

**Table 5:** The fermentation performances of *P. stipitis* wild strain and TJ2-3 using fermentation media of three sugar types at 30°C

	*P. stipitis* TJ2-3			Wild strain		
Parameter	Xylose	Glucose	Mix	Xylose	Glucose	Mix
Total sugar [%(w/v)]	12.4 ± 0.4	12.4 ± 0.4	12.4 ± 0.4	12.4 ± 0.4	12.4 ± 0.4	12.4 ± 0.4
Residual sugar [%(w/v)]	1.1 ± 0.2	0.5 ± 0.4	5.8 ± 0.3	3.9 ± 0.2	3.5 ± 0.2	6.1 ± 0.1
Ethanol [%(w/v)]	4.1 ± 0.3	4.1 ± 0.7	3.7 ± 0.1	2.9 ± 0.3	3.5 ± 0.1	2.4 ± 0.3
Yield (total sugar) (%)	68.3 ± 0.7	73.8 ± 0.2	61.6 ± 0.8	49.9 ± 0.8	54.8 ± 0.7	42.9 ± 0.3
Yield (consumed sugar) (%)	75.8 ± 1.2	77.9 ± 0.6	87.0 ± 0.7	72.1 ± 1.4	77.9 ± 0.5	75.7 ± 0.9
Fermentation time (h)	96	96	96	96	96	96
Maximum productivity (g l^−1^ h^−1^)	0.6 ± 0.0	0.9 ± 0.1	0.6 ± 0.1	0.5 ± 0.0	0.9 ± 0.1	0.6 ± 0.0

Results are means ± SD for three independent experiments.

Fermentation of the wild-type and TJ2-3 strains of *P. stipitis* using mixed sugars yielded identical results. The phenomenon called ‘glucose repression’ has been previously reported, in which glucose represses existing xylose fermentation. In the present study, the repression of xylose fermentation by glucose was responsible for the observed differences between TJ2-3 and the wild-type. Thus, when glucose was completely exhausted, no more ethanol was produced from xylose.

The results of carbon balances in recombinant *S. cerevisiae* calculated from the initial HPLC data in the previous studies showed that 11% of the carbon on xylose and 3% of the carbon on glucose were undetectable (Kuyper *et al*., 2004). Given that *S. cerevisiae* uses xylose more slowly than glucose, relatively high amounts of ethanol evaporated when xylose was used. If the pentose metabolic pathway in *P. stipitis* was considered, the recombinant *S. cerevisiae* could utilize xylose at a low rate and accumulate xylitol as main by-product. On the contrary, *P. stipitis* could directly ferment xylose to ethanol with few by-products. The different patterns of xylose metabolism and NADPH generation provide some information on the fermentation performances of *P. stipitis* and the recombinant *S. cerevisiae* (Guo and Jiang, 2009).

Some studies have been conducted on the production of ethanol from xylose by engineered *S. cerevisiae*, which expresses xylose isomerase (Kim *et al*., 2012; Lee *et al*., 2012) or XR-XDH recombinant *S. cerevisiae* strains (Chu and Lee, 2007; Hahn-Hägerdal *et al*., 2009). The results showed that utilization of XR-XDH in the xylose metabolic pathway is more efficient for ethanol production. Therefore, the XR-XDH pathway in *P. stipitis* attracted more attention.

### Mechanisms of improved ethanol production in the mutant strain

**Figure 3:**
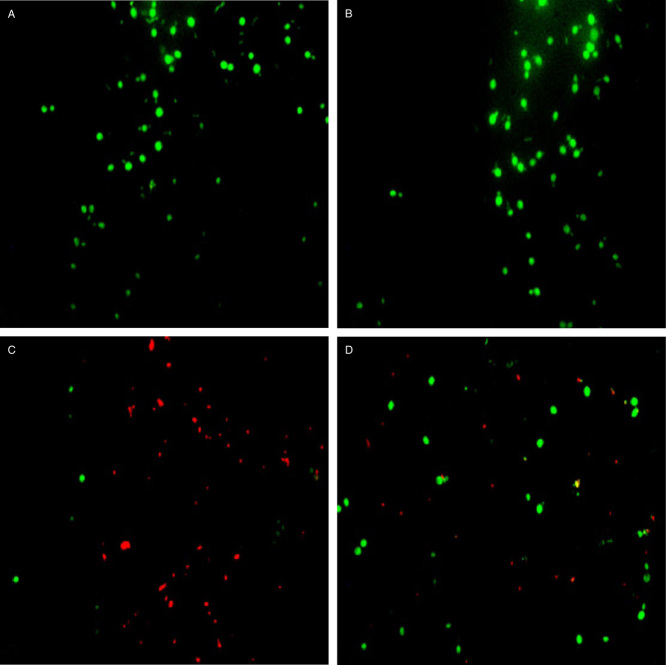
Representative images of living (green), apoptotic (yellow) and necrotic (red) cells stained with AO/PI before and after fermentation. Wild-type strain before (A) and after (C) fermentation. TJ2-3 strain before (B) and after (D) fermentation.

The cell viability was measured based on the number of AO- and PI-positive cells in the samples. The staining results are shown in Fig. 3. The accumulation of PI-positive cells occurred when the yeast cultures were allowed to continue fermentation over 96 h. The wild-type *P. stipitis* had higher levels of PI-positive cells than the mutant strain (TJ2-3; Fig. 3A and B). The mutant strain TJ2-3 was more capable of maintaining membrane integrity.

The enhanced ethanol tolerance of a strain is important for industrial ethanol fermentation. The viability of the parent and mutant strains was measured using their exposure to different concentrations of additional ethanol. All mutant strains demonstrated their considerably improved tolerance to ethanol stress (data not shown). When the concentration of additional ethanol concentration was increased to 10% (v/v), the cell viability was decreased in all strains. However, the mutant strains were noticeably more resistant to ethanol stress than the wild-type strain. During the next round, the mutant strains showed higher viability than those from the former rounds of shuffling (data not shown). When the concentration of additional ethanol was increased to 13% (v/v), the TJ2-3 strain clearly demonstrated cell viability, whereas the other cells lost their viability (data not shown).

### Chromosome-spreading assay

**Figure 4:**
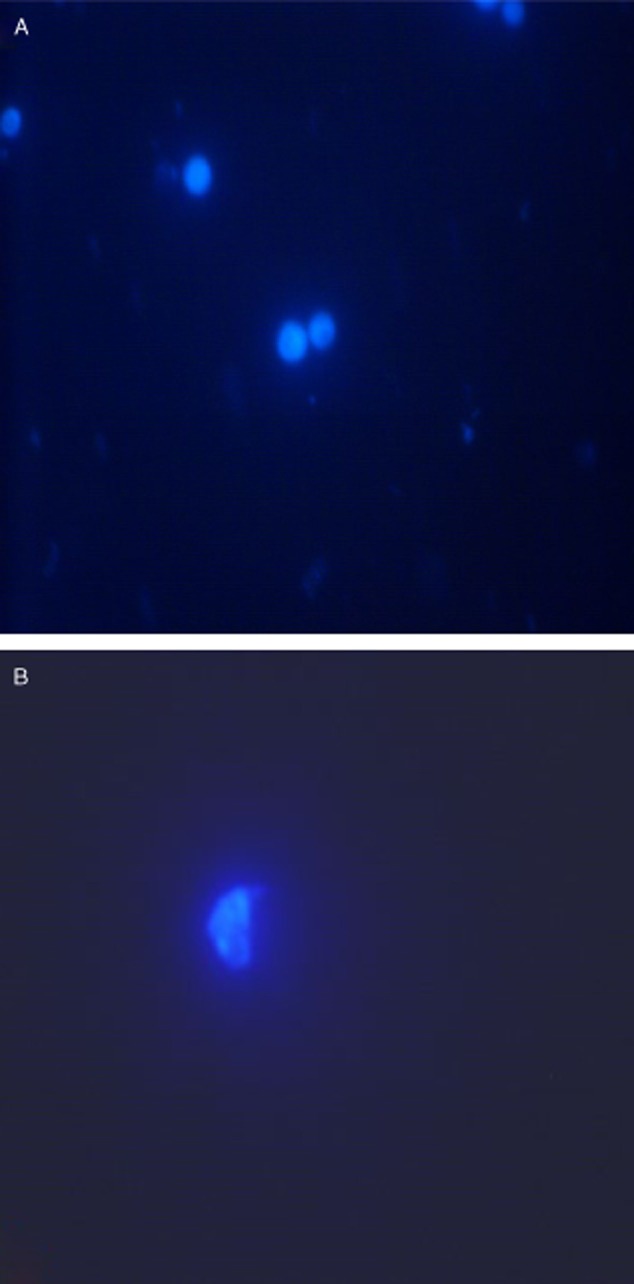
Increased DNA instability in cells visualized by DAPI staining. Nuclei were stained with DAPI (blue). Images were photographed with the same exposure time using a Nikon microscope equipped with a cooled CCD camera under 60 × magnification.

Chromosomal aberrations or DNA mutations demonstrate the cytological phenotype of the mutations caused by high-energy-pulse electron beams in previous studies (Zhang *et al*., 2012b). Thus, chromosome analysis is considered a necessary tool for analysing yeast mutants. To detect DNA instability, inactivated cells were lysed on a glass slide plate, and their chromosomal DNA was stained with DAPI. Cells that were inactivated by the combined UV and LiCl treatment (Fig. 4B) exhibited increased chromosome aneuploidy, as compared with the control cells (Fig. 4A). Thus, the yeast cell mutant strain was a consequence of the induced DNA and genetic instability.

### Genetic stability of the mutant strain

The mutant strain (TJ2-3) was cultured for 50 generations, and the resulting ethanol production was measured by HPLC. The results showed that the ethanol production from TJ2-3 did not decrease over time, thereby illustrating that the strain was genetically stable.

### Enzymatic analysis of the mutation *P. stipitis* and wild strain

During xylose fermentation, the wild and TJ2-3 strains exhibited a significant NADH-linked XR activity (Table 6), which is the key to xylose fermentation by yeasts (Lee *et al*., 2003). Compared with the wild strain, the TJ2-3 strain displayed higher XR activity (0.642 vs. 0.967). In sum, the mutant TJ2-3 strain showed increased pleiotropic activities of XR (51%), XDH (32%) and glucose-6-phosphate dehydrogenase (20%). Therefore, the increased ethanol production from xylose fermentation by the mutant strains, as well as the increased pleiotropic activities of several enzymes (Table 6), indicate the existence and participation of the putative regulatory gene in the synthesis of several enzymes for xylose metabolism.

**Table 6:** Enzyme activities in the cell extracts of the *Pichia stipitis* ATCC 5836 and the mutant strains TJ2-3

	Enzyme					
Strain	Xylose reductase	Xylitol dehydrogenase	Glucose-6-phosphate dehydrogenase	Hexokinase	Glyceraldehyde-3-phosphate dehydrogenase	Alcohol dehydrogenase
ATCC 5836	0.642 ± 0.115	1.011 ± 0.465	0.423 ± 0.056	0.023 ± 0.001	0.125 ± 0.012	0.241 ± 0.064
TJ2-3	0.967 ± 0.108	1.334 ± 0.063	0.508 ± 0.061	0.031 ± 0.004	0.141 ± 0.006	0.265 ± 0.015

The activity units: U mg^−1^ protein (*U* unit: μmoles × mg protein^−1^ × min^−1^).

Ethanol has been known to be an important repressor that prevents the activities of specific enzymes needed for xylose utilization (Slininger *et al*., 2011). When the ethanol concentration was increased, the XR and XD activities were almost completely blocked, which delayed or stalled the ethanol fermentation. This observation can be a defence mechanism to avoid further cell damage and conserve energy. Low XR and XD activities, which were enough to allow a very slow xylose metabolism rate in producing a very low (or no) ethanol, were observed. Given the comparable fermentation behaviour of the two strains, the specific XR and XDH activities in the cell extracts of the two strains were tested. The different patterns of xylose consumption and ethanol generation showed the increased enzyme activity caused by genome shuffling. The activity of XR and XDH in TJ2-3 is much higher than that in the wild strain (Table 6). Understanding the mechanism of xylose-specific enzyme activities in *P. stipitis* is useful for determining a metabolic engineering strategy to increase the production of ethanol from xylose using *P. stipitis*.

## Conclusions

In this study, we used a modified genome shuffling method to rapidly screen for a xylose-fermenting *P. stipitis* strain. In combination with an appropriately designed screening strategy, the mutant TJ2-3 strain was obtained. This yeast mutant had enhanced rates of xylose consumption and ethanol production, as compared with the parental strains. Combined with traditional screening, genome shuffling is highly efficient for generating strains with desired phenotypes. The molecular mechanisms involved still need to be further studied before the commercial use of these developed industrial *P. stipitis* strains with the enhanced capacity to ferment xylose into ethanol.

## Experimental procedures

### Strain, media and growth conditions

The *P. stipitis* strain ATCC58376 was obtained from China General Microbiological Culture Collection Center and maintained in yeast peptone-xylose (YPX) liquid medium, which contained 1% yeast extract, 2% peptone and 2% xylose (w/v). The xylose fermentation medium (pH 5.5) was composed of 5–12% xylose, 0.5% yeast extract, 0.5% KH_2_PO_4_, 0.2% (NH_4_)_2_SO_4_, 0.02% CaCl_2_ and 0.02% MgSO_4_·7H_2_O (w/v). The recombinant strains were selected on yeast nitrogen base (YNB) with 5% xylose (YNBX) plates at 35°C for 2 days.

### Protoplast preparation and regeneration

*Pichia stipitis* cells were harvested by centrifugation at 4000 × *g* for 5 min at 4°C. The cells were washed twice with PB buffer (0. 01 M Tris–HCl, pH 6.8; 20 mM MgCl_2_; and 0.5 M sucrose as a stabilizer, pH 7.0), and resuspended in a mixture of 1% (w/v) β-mercaptoethanol and 2% (w/v) snail enzyme. The protoplasts were obtained by incubating the suspension at 30°C for 60 min. The cells were again centrifuged and resuspended in the protoplast preparation agent (Shi *et al*., 2002). The rates of protoplast formation and regeneration were obtained from colony counts using the following formulas:


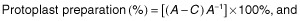



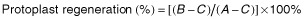


where *A* refers to the total number of colonies counted on the YPX medium before cell wall hydrolysis by the snail enzyme, *B* represents the number of colonies counted on the regeneration medium (RM) agar (the cells with enzyme-treated were spread on the RM agar), and *C* represents the total number of colonies counted on YPX medium after cell wall hydrolysis by the snail enzyme.

### Protoplast inactivation

The prepared protoplasts (1.0 × 10^7^ cells ml^−1^) were resuspended in 5 ml of the protoplast preparation agent. A final concentration of 0.6% (w/v) LiCl was introduced to the protoplast suspension. The suspensions were irradiated for 5, 10, 15, 20, 25 or 30 min under a preheated 30 W UV lamp at a vertical distance of 20 cm to select for the optimal inactivation condition. The exposed protoplasts were kept in the dark for 2 h to avoid light repair. Cells inactivated by the combination of UV and LiCl were verified by the lack of clones growing on the YPX medium.

### Genome shuffling

Protoplasts from the seed culture of *P. stipitis* were inactivated by heating or by exposure to UV with or without LiCl. The inactivated protoplasts were divided into two groups. The protoplasts were randomly fused and regenerated under suitable conditions. Protoplast fusion was observed by confocal laser-scanning microscopy (CLSM). All regenerated colonies were collected and selected using the YPX medium as the preliminary screening method. This step corresponded to the first round of genome shuffling. The selected colonies were referred to as the first generation, which were used as the new parental strains during the second round of genome shuffling. During this second round, the same conditions for protoplast preparation and inactivation were used. The target strain was obtained after two rounds of genome shuffling.

### Shake-flask fermentation

Cultures inoculated with the mutant *P. stipitis* were added with 120 g l^−1^ xylose and fermented at 30°C, with a shaking speed of 200 r.p.m. for 96 h. The fermentation broth of each flask was serially diluted by 100-fold. A 1 ml sample of each culture was placed in a 1.5 ml centrifuge tube and centrifuged at 13 000 r.p.m. for 10 min. The fermented cultures were subsequently analysed by high-performance liquid chromatography (HPLC). The glucose concentration was determined using an Aminex HPX-87H column (Bio-Rad, Richmond, CA, USA) at 65°C, using 5 mM H_2_SO_4_ as the eluent at a rate of 0.6 ml min^−1^. HPLC results were obtained using a differential refractive index detector. The yeast cell concentration was based on the optical density at 600 nm (OD_600_), as measured by a spectrometer. An OD_600_ of 1 for the *P. stipitis* cell suspension was equivalent to approximately 0.32 g dry cells l^−1^. The ethanol productivity was measured by the following equation:


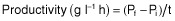


where P_i_ is the initial ethanol concentration (g l^−1^), P_f_ is the final ethanol concentration (g l^−1^), and t is the fermentation time (h).

The maximum theoretical ethanol yield from glucose or xylose was measured using the following relation: 100 g of glucose/xylose produces 51.1 g of ethanol and 48.9 g of CO_2_.

Ethanol yields were calculated using the following equation:


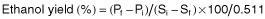


where P_i_ is the initial ethanol concentration (g l^−1^), P_f_ is the final ethanol concentration (g l^−1^), S_i_ is the initial sugar concentration (g l^−1^) and S_f_ is the final sugar concentration (g l^−1^).

### Cell viability and membrane integrity by fluorescence microscopy

To analyse the cell viability and membrane integrity, samples were stained with 2 μl of an acridine orange (AO)–propidium iodide (PI) solution (1:1, v/v). Staining was performed at 37°C for 15 min in the dark, and the cells were subsequently examined via a fluorescence microscope. Under blue light, the necrotic cells were stained red, whereas the apoptotic cells were bright orange or yellow.

### Chromosome-spreading assay

The yeast protoplasts in LiCl were exposed to UV light for 0 or 10 min and subsequently placed on a glass plate. Chromatin was simultaneously spread and fixed by treating the glass plate first with 25 μl 4% paraformaldehyde with 3.4% sucrose, followed by 40 μl 1% Triton X-100, and finally with 80 μl 4% paraformaldehyde containing 3.4% sucrose. The fixed DNA was stained with 4′,6-diamido-2-phenylindole (DAPI). DAPI is excited in near-UV light at 365 nm, and its emission is within the violet-blue spectral range. DAPI staining revealed the locations of cell nuclei, particularly the chromosomes therein. Fluorescence images were acquired using a Nikon microscope equipped with a cooled charge-coupled device camera.

### Genetic stability of the mutant strain

The *P. stipitis* mutant strain with the highest ethanol production was cultured for 50 generations. Based on the results of gas chromatography analysis, the fermentation product contained similar levels of ethanol as the starting inoculum of the mutant strains. Thus, the isolated mutant was considered to be genetically stable.

### Enzymatic activity analysis

The cells were harvested by centrifugation at 5000 r.p.m. min^−1^ for 5 min and washed twice with ice-cold sterile water. Then the cells were resuspended in 100 mM potassium phosphate buffer containing 2 mM MgCl_2_ and 2 mM DTT, and mixed with glass beads by vortexing for 30 min. The cells were centrifuged at 13 000 r.p.m. min^−1^ for 60 min. All the cell crude extract was kept on ice. The units were normalized by the amount of total protein (mg) measured by a BCA (bicinchonininc acid) protein assay kit.

Xylose reductase (XR) activities were measured in 1 ml of reaction solution, including 0.7 ml phosphate buffer (50 mM), 0.1 ml cell extracts, 0.1 ml NADPH(2 mM) and 0.1 ml xylose(1 M). The solution without xylose was incubated for equilibrium (1 min at 25°C). After adding xylose for 2 min, the reaction solution was measured at 340 nm by a spectrophotometer. Xylitol dehydrogenase (XDH) activities were also measured as above. The1 ml of reaction solution including 0.7 ml Tris buffer (50 mM), 0.1 ml the cell extracts, 0.1 ml NAD^+^(2 mM) and 0.1 ml xylitol (1 M). One unit (1 U) of enzyme was defined by the conversion of 1 μmol NADPH (XR) or NAD^+^ (XDH) per minute.
